# Enhanced gyrotropic birefringence and natural optical activity on electromagnon resonance in a helimagnet

**DOI:** 10.1038/s41467-021-26953-x

**Published:** 2021-11-18

**Authors:** S. Iguchi, R. Masuda, S. Seki, Y. Tokura, Y. Takahashi

**Affiliations:** 1 Department of Applied Physics and Quantum Phase Electronics Center (QPEC), University of Tokyo, Tokyo, 113-8656 Japan; 2 Institute of Engineering Innovation, University of Tokyo, Tokyo, 113-0032 Japan; 3 RIKEN Center for Emergent Matter Science (CEMS), Wako, Saitama, 351-0198 Japan; 4 Tokyo College, University of Tokyo, Tokyo, 113-8656 Japan

**Keywords:** Ferroelectrics and multiferroics, Terahertz optics

## Abstract

Spontaneous symmetry breaking in crystalline solid often produces exotic nonreciprocal phenomena. As one such example, the unconventional optical rotation with nonreciprocity, which is termed gyrotropic birefringence, is expected to emerge from the magnetoelectric coupling. However, the fundamental nature of gyrotropic birefringence remains to be examined. Here w`e demonstrate the gyrotropic birefringence enhanced by the dynamical magnetoelectric coupling on the electrically active magnon resonance, i.e. electromagnon, in a multiferroic helimagnet. The helical spin order having both polarity and chirality is found to cause the giant gyrotropic birefringence in addition to the conventional gyrotropy, i.e. natural optical activity. It is demonstrated that the optical rotation of gyrotropic birefringence can be viewed as the nonreciprocal rotation of the optical principal axes, while the crystallographic and magnetic anisotropies are intact. The independent control of the nonreciprocal linear (gyrotropic birefringence) and circular (natural optical activity) birefringence/dichroism paves a way for the optically active devices.

## Abstract (web summary)

Gyrotropic birefringence is expected to emerge from the magnetoelectric coupling. Here, the authors find polarity and chirality arising from the helical spin order exhibit enhanced optical activities, gyrotropic birefringence and natural optical activity, on the electromagnon resonance.

## Introduction

The nonreciprocity of quantum particles such as a photon, magnon, and conduction electron has been observed in a variety of quantum materials including the multiferroics, polar semiconductors, Weyl semimetal, and noncentrosymmetric superconductors^1–5^, in which the sign of the momentum, +*k*^*ω*^ or −*k*^*ω*^, causes the nonreciprocity for the physical properties of these particles. While such responses are allowed in any medium with broken symmetries of space inversion and time reversal, the spontaneous symmetry breaking enables to cause the extremely enhanced nonreciprocal responses, which potentially provides the device functionality. The nonreciprocity of photon is observed as the different optical responses for the counter-propagating lights having the opposite momentum, +*k*^*ω*^ or −*k*^*ω*^. For example, the giant nonreciprocal photon absorption, i.e., directional dichroism, has been demonstrated in the multiferroics with the ferroelectric and long-range magnetic orders^6–9^. In addition, the polarization degrees of freedom of photon can also exhibit the nonreciprocity, that is the gyrotropic birefringence (GB), producing the opposite polarization rotation for the counter-propagating lights. The GB is expected to realize in various magnetoelectric (ME) or multiferroic materials^10–14^, and the resulting enhanced exotic optical rotation leads to the control of light polarization.

**Fig. 1:**
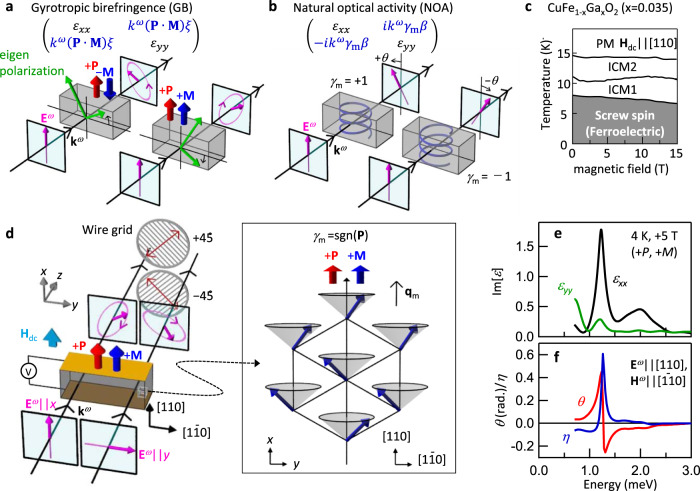
Gyrotropic birefringence (GB) and natural optical activity (NOA) on electromagnon in helimagnet CuFe_1-x_Ga_x_O_2_ (x = 0.035). **a** The ferroelectric polarization **P** parallel or antiparallel to the magnetization **M** induces the GB. The GB response can be viewed as the rotation of eigen polarization (green arrows). The reversal of either **P** or **M** reverses the sign of the rotation of eigen polarization. The nonreciprocal optical rotation of GB is represented by the symmetric off-diagonal elements in the effective dielectric tensor. **b** Schematics of the optical rotation by NOA. The chiral media show the optical rotation owing to the circular birefringence/dichroism, whose sign depends on the sign of chirality$\,{\gamma }_{{{{{{\rm{m}}}}}}}$. The antisymmetric parts of the effective dielectric tensor represent the NOA. **c** Magnetic phase diagram of CuFe_1-x_Ga_x_O_2_ (x = 0.035) in the external magnetic field H_dc_||[110]||${{{{{{\bf{q}}}}}}}_{{{{{{\rm{m}}}}}}}$^29^. **d** The schematics of terahertz time-domain polarimetry and of conical screw spin structure in the magnetic field H_dc_||[110]. The sign of chirality (${\gamma }_{{{{{{\rm{m}}}}}}}$) is rigidly connected to that of **P** as $\,{\gamma }_{{{{{{\rm{m}}}}}}}={{{{{\rm{sgn}}}}}}({{{{{\bf{P}}}}}})$^29^. **e** Imaginary parts of ${\varepsilon }_{xx}$ and ${\varepsilon }_{yy}$ in the magnetic field (5 T) at 4 K. **f** Rotation angle *θ* and ellipticity *η* for the incident light with E^*ω*^||[110]. The sample thickness is 1 mm.

The optical rotation stemming from GB is essentially different from the conventional polarization rotation phenomena such as the magneto-optical Faraday effect and the natural optical activity (NOA), in which the circular birefringence/dichroism are responsible for the polarization rotation. From the viewpoint of the inherent response of matter itself, the NOA is described by the *k*^*ω*^-dependent, i.e., nonreciprocal, optical rotation, whereas the magneto-optical effects preserve the reciprocity. Thus, there are two different types of nonreciprocal optical rotations, GB and NOA. It is suggested that the GB can be viewed as the *k*^*ω*^-dependent rotation of the optical principal axes of matter^10^. Thus, the optical rotation of GB is characterized by the nonreciprocal linear birefringence/dichroism, where the linear polarization is eigen polarization (Fig. 1a). The change of eigen polarization by GB results in the optical rotation. Note that the crystallographic anisotropy, which is the usual origin of reciprocal linear birefringence/dichroism, is kept intact even when the GB emerges. This optical property of GB is contrasted with the NOA, whose eigen polarization is circular polarization. The existence of GB was initially suggested by the small polarization rotation in the ME antiferromagnet Cr_2_O_3_^11^. So far, the optical rotation observed in a few materials has been ascribed to GB^11,12,15–17^, but such a remarkable nature of GB has not been examined and remains elusive.

The dynamical ME coupling is the main cause of nonreciprocal optical processes in matter^10,18^. The breaking of both time-reversal and space-inversion symmetries induces the linear ME coupling described in terms of ${\alpha }_{ij}{E}_{i}{H}_{j}$ in the free energy formula, where the electric field ${E}_{i}$ and magnetic field ${H}_{j}$ are coupled through the ME tensor ${\alpha }_{ij}$ (*i, j* = *x*, *y*, *z*)^19^. Accordingly, the magnetization **M** (or polarization **P**) is produced by the electric field **E** (or magnetic field **H**) through this ME coupling as ${M}_{j}={\alpha }_{ij}{E}_{i}$ (or ${P}_{i}={\alpha }_{ij}{H}_{j}$), resulting in the modified Maxwell’s equations in ME media. The light–matter interaction including the dynamic ME coupling ${\alpha }_{ij}(\omega )$ produces the nonreciprocal optical effects, in which both electric (**E**^*ω*^) and magnetic (**H**^*ω*^) field of light are responsible for the optical processes. The off-diagonal terms, ${\alpha }_{ij}(\omega )\,(i\ne j)$, give rise to the directional dichroism while keeping the light eigen polarization, as exemplified by many ME multiferroics^6–9,18^. On the other hand, the diagonal ME coupling (${\alpha }_{ii}(\omega )$), which induces the magnetization along the applied electric field, is supposed to cause the nonreciprocal polarization rotation, i.e., GB^10–12^. Since this ME coupling induces the electric polarization ($\varDelta {{{{{{\bf{P}}}}}}}^{\omega }=\alpha {{{{{{\bf{H}}}}}}}^{\omega }$) being normal to the electric field of light as $\varDelta {{{{{{\bf{P}}}}}}}^{\omega }{{{{{{\bf{E}}}}}}}^{\omega }$, the light polarization is always modified while the light passes through the matter. In addition to the multiferroics, a similar ME coupling emerges in the ME monopole embedded in condensed matter and in the axion electrodynamics of topological materials^20–22^.

To approach the nature of GB, we focus on the strong dynamical ME coupling of the electromagnon resonance in the terahertz region, which is the magnon excitation accompanied by the magnetic and ferroelectric orders^8,23,24^. The coexistence of spin-driven polarity and chirality in helical spin order gives rise to the GB and NOA simultaneously, enabling the comparative study of these different optical activities^25^. We exploit the phase-sensitive terahertz time-domain polarimetry, which can provide the representation of optical response in the form of an effective dielectric tensor. Such a matrix spectral analysis enables the clear distinction of GB and NOA, establishing the basis of nonreciprocal photonic responses.

## Results

The screw-type helical spin order in archetypal multiferroic CuFe_1-x_Ga_x_O_2_ (x = 0.035), in which the slight Ga-doping stabilizes the helical spin phase, exhibits the spin-driven ferroelectricity and chirality^26–29^. The crystal structure in the paramagnetic phase belongs to the centrosymmetric point group (*R*$\bar{3}$*m*). The frustration of spin interactions among Fe^3+^ sites gives rise to the successive magnetic phase transitions from a paramagnetic phase (PM) to two incommensurate collinear spin phases (ICM1 and ICM2) and the noncollinear screw spin phase with ferroelectricity (Fig. 1c)^26,27^. The screw spin phase is characterized by the magnetic modulation vector (*q*_m_, *q*_m_, 3/2) with *q*_m_ = 0.202 (Fig. 1d)^28^. To refer to the modulation vector, hereafter, we omit the out-of-plane component of the magnetic modulation vector as **q**_m_ = (*q*_m_, *q*_m_, 0) for brevity. The screw spin order gives rise to the spontaneous polarization **P** along the screw axis (**q**_m_ | |[110], Fig. 1d), which is explained by the *d*-*p* orbital hybridization mechanism^27–30^. In the external magnetic field (**H**_dc_ | |**P**), the proper screw spin structure continuously transforms into the longitudinal conical spin structure, resulting in **M** | | **P** (Fig. 1d). This geometry allows the diagonal ME coupling (${\alpha }_{ii}\ne 0$) and hence the GB (Fig. 1a). In addition, the right-handed and left-handed screw habits of the spin order has the magnetically-induced chirality, producing the NOA (Fig. 1b)^25^. The sign of chirality (*γ*_m_ = ±) is rigidly connected to the sign of **P** as *γ*_m_ = sgn(**P**).

The terahertz time-domain polarimetry was employed to measure the optical rotations caused by GB and NOA. Hereafter, we use the experimental coordinates *x*, *y*, and *z* for [110], [1$\bar{1}$0], and [001] (Fig. 1d), respectively. The polarization rotation is analyzed by using the wire grid polarizer set to ±45° with respect to the *x* axis, providing the full polarization states of light including phase and amplitude. Figure 1e shows the terahertz spectra for *ε*_*xx*_ and *ε*_*yy*_ in the magnetic field (+5 T, **H**_dc_ | |*x*). Clear resonances at 1.2 and 2.0 meV are observed for Im[*ε*_*xx*_] (black), while little resonance structure is discerned for Im[*ε*_*yy*_] (green). Although the (001) plane is isotropic in the paramagnetic phase, the screw spin order with in-plane screw axis (**q**_m_ | |*x*) induces the anisotropic optical responses between *ε*_*xx*_ and *ε*_*yy*_. The large optical rotation *θ*, which is as large as 0.4 rad (∼23°) for the sample thickness of 1 mm, was observed in the screw spin phase (Fig. 1f). The rotation angle *θ* and the ellipticity *η* show the clear dispersive and peaking structures at 1.2 meV, respectively, indicating that the enhanced optical rotation emerges on the lower-lying electromagnon resonance. It should be noted that the magneto-optical Faraday and Kerr rotations are prohibited in this Voigt geometry (Fig. 1d).

**Fig. 2:**
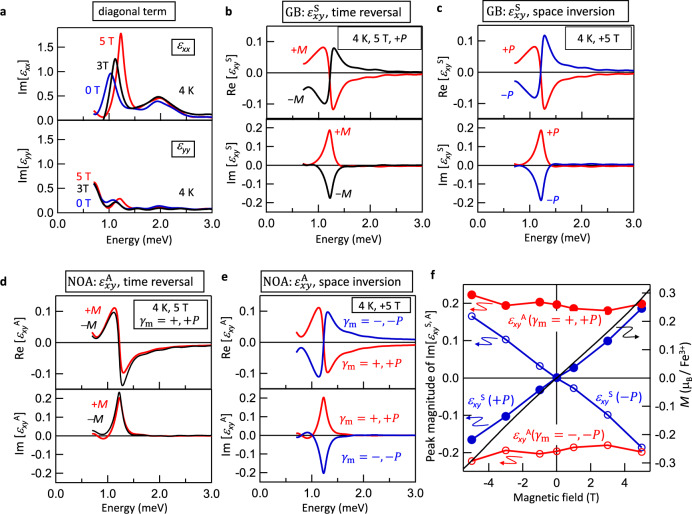
Spectra of GB and NOA with response to **P**, **M**, and ${\gamma }_{{{{{{\rm{m}}}}}}}$. **a** Magnetic field dependence of the diagonal parts of effective dielectric tensor (${\varepsilon }_{xx}$ and ${\varepsilon }_{yy}$) at 4 K in the magnetic field (**H**_dc_|| [110]). Symmetric part of effective dielectric spectra (${\varepsilon }_{xy}^{{{{{{\rm{S}}}}}}}$, see Eq. (1)) representing the optical rotation of GB under **b** time reversal and **c** space inversion. Antisymmetric part of effective dielectric spectra (${\varepsilon }_{xy}^{{{{{{\rm{A}}}}}}}$) representing the optical rotation of NOA under **d** time reversal and **e** space inversion. The magnetically-induced chirality *γ*_m_ = ±, and equivalently the sign of **P**, are denoted. **f** Magnetic field dependence of the peak magnitude of Im[${\varepsilon }_{xy}^{{{{{{\rm{A}}}}}}}$] (red) and Im[${\varepsilon }_{xy}^{{{{{{\rm{S}}}}}}}$] (blue) and the magnetization *M* (black line) of CuFe_1-x_Ga_x_O_2_ (x = 0.035) (right axis)^29^. The filled and open circles denote the peak magnitude for (*γ*_m_ = +, +**P**) and (*γ*_m_ = −, −**P**) states, respectively. (See Supplementary Note 2 for details of spectra).

To examine the spectral characteristics of GB, we approximately express the optical rotation of GB and NOA by exploiting the effective dielectric tensor. In addition to the diagonal spectra *ε*_*xx*_ and *ε*_*yy*_ (Fig. 2a), the complex spectra of off-diagonal elements, *ε*_*xy*_ and *ε*_*yx*_, can be obtained by the terahertz time-domain polarimetry (Supplementary Note 1). We analyzed the observed off-diagonal spectra, which represent the optical rotation, on the basis of the following assumptions. If the GB can be viewed as the nonreciprocal linear birefringence, the symmetric part of the off-diagonal term ${\varepsilon }_{xy}^{{{{{{\rm{S}}}}}}}=\frac{{\varepsilon }_{xy}+{\varepsilon }_{yx}}{2}$ represents the optical rotation induced by GB^10^. The GB arising from the ME coupling should be odd under the reversal of either of *k*^*ω*^, ${{{{{\bf{P}}}}}}$, or **M**, so that the leading order of the expansion of ${\varepsilon }_{xy}^{{{{{{\rm{S}}}}}}}$ is expressed as ${k}^{\omega }({{{{{\bf{P}}}}}}\cdot {{{{{\bf{M}}}}}})\beta$, where $\beta$ is an arbitrary constant (Fig. 1a). On the other hand, the optical rotation of NOA is described by the antisymmetric dielectric tensor $i{\varepsilon }_{xy}^{{{{{{\rm{A}}}}}}}=\frac{{\varepsilon }_{xy}-{\varepsilon }_{yx}}{2}$, leading to the circular birefringence and dichroism^19,25^. For the NOA, the ${\varepsilon }_{xy}^{{{{{{\rm{A}}}}}}}$ should be odd under the reversal of *k*^*ω*^ or chirality ${\gamma }_{{{{{{\rm{m}}}}}}}$, but even under the reversal of **M**, so that ${\varepsilon }_{xy}^{{{{{{\rm{A}}}}}}}$ is expressed as *k*^*ω*^${\gamma }_{{{{{{\rm{m}}}}}}}\beta$, where $\beta$ is an arbitrary constant (Fig. 1b). Therefore, the effective dielectric tensor representing the GB and NOA is described as follows,

1 $$\left(\begin{array}{cc}{\varepsilon }_{xx} & {\varepsilon }_{xy}\\ {\varepsilon }_{yx} & {\varepsilon }_{yy}\end{array}\right)=\left(\begin{array}{cc}{\varepsilon }_{xx} & {\varepsilon }_{xy}^{{{{{{\rm{S}}}}}}}+i{\varepsilon }_{xy}^{{{{{{\rm{A}}}}}}}\\ {\varepsilon }_{xy}^{{{{{{\rm{S}}}}}}}-i{\varepsilon }_{xy}^{{{{{{\rm{A}}}}}}} & {\varepsilon }_{yy}\end{array}\right) \sim \left(\begin{array}{cc}{\varepsilon }_{xx} & {k}^{\omega }({{{{{\bf{P}}}}}}\cdot {{{{{\bf{M}}}}}})+i{k}^{\omega }{\gamma }_{{{{{{\rm{m}}}}}}}\beta \\ {k}^{\omega }({{{{{\bf{P}}}}}}\cdot {{{{{\bf{M}}}}}})-i{k}^{\omega }{\gamma }_{{{{{{\rm{m}}}}}}}\beta & {\varepsilon }_{yy}\end{array}\right).\,$$

Note that the diagonal ME coupling ${\alpha }_{ii}$ and magnetic permeability ${\mu }_{ij}$, which are responsible for the GB and NOA as discussed later, are implicitly included in the effective dielectric tensor. The observed spectra at 5 T are summarized in Fig. 2. Both symmetric part ${\varepsilon }_{xy}^{{{{{{\rm{S}}}}}}}$ (Fig. 2b, c) and antisymmetric part ${\varepsilon }_{xy}^{{{{{{\rm{A}}}}}}}$ (Fig. 2d, e) exhibit the clear resonance structure on the lower-lying electromagnon at 1.2 meV (5 T in Fig. 2a).

The symmetry of ${\varepsilon }_{xy}^{{{{{{\rm{S}}}}}}}$ and ${\varepsilon }_{xy}^{{{{{{\rm{A}}}}}}}$ under space inversion or time reversal can be examined by the reversal of **P** or **M**, respectively. For the ${\varepsilon }_{xy}^{{{{{{\rm{S}}}}}}}$ (Fig. 2b, c), the sign reversal of the resonance peak is clearly observed. The positive peak structure for +**M** (red) turns into the negative one (black) by the reversal of **M** (Fig. 2b). The spectrum for +**P** (red) also shows the sign reversal under the reversal of **P** (blue spectrum in Fig. 2c). Therefore, it is concluded that the ${\varepsilon }_{xy}^{{{{{{\rm{S}}}}}}}$ is odd under both time reversal and space inversion as expected for GB. On the other hand, the ${\varepsilon }_{xy}^{{{{{{\rm{A}}}}}}}$ representing NOA preserves the sign of resonance peak under the reversal of **M** (Fig. 2d). The sign change of ${\varepsilon }_{xy}^{{{{{{\rm{A}}}}}}}$ is induced by the reversal of magnetically-induced chirality (${\gamma }_{{{{{{\rm{m}}}}}}}$) (Fig. 2e), or equivalently the reversal of **P**, indicating the essential role of chirality for NOA. Thus ${\varepsilon }_{xy}^{{{{{{\rm{A}}}}}}}$ is odd under space inversion, while being even under time reversal, as expected for the symmetry characteristics of NOA.

The magnetic field dependence of peak intensities for Im[${\varepsilon }_{xy}^{{{{{{\rm{S}}}}}}}$] (GB) and Im[${{\varepsilon} _{xy}^{{{{{\rm{A}}}}}}}$] (NOA) clearly exemplifies the above symmetry characteristics (Fig. 2f). The **M** is almost proportional to the magnetic field (**H**_dc_), while the **P** is intact in the magnetic field below 5 T^28,29^. The peak magnitude of ${\varepsilon }_{xy}^{{{{{{\rm{S}}}}}}}$ (GB, blue circles) is proportional to **H**_dc_ and shows the sign change for reversal of **P**. Accordingly, the leading order of expansion of ${\varepsilon }_{xy}^{{{{{{\rm{S}}}}}}}$ is suggested to be proportional to ${{{{{\bf{P}}}}}}\cdot {{{{{\bf{M}}}}}}$. These results corroborate the validity of the above assumption in Eq. (1), in which the GB is expressed as ${\varepsilon }_{xy}^{{{{{{\rm{S}}}}}}} \sim {k}^{\omega }({{{{{\bf{P}}}}}}\cdot {{{{{\bf{M}}}}}}).$ In contrast, ${\varepsilon }_{xy}^{{{{{{\rm{A}}}}}}}$ (NOA, red circles) shows little **H**_dc_ dependence (Fig. 2d), whereas the sign change is observed for the reversal of chirality (*γ*_m_ = ±). Thus, the ${\varepsilon }_{xy}^{{{{{{\rm{A}}}}}}}$ can be described by ${k}^{\omega }{\gamma }_{{{{{{\rm{m}}}}}}}\beta$ as expected generically for the optical response of chiral matter.

According to the above results, it is explicitly concluded that the GB can be interpreted as the linear birefringence/dichroism (${\varepsilon }_{xy}^{{{{{{\rm{S}}}}}}}$) induced by the rotation of the eigen polarization (Fig. 1a). In addition, the (anti-)symmetrized off-diagonal spectra enable us to distinguish the optical response of GB from the conventional gyrotropy qualitatively and quantitatively. It should be emphasized that although the usual crystallographic and magnetic anisotropy axis (||*x* and **q**_m_) is preserved in the magnetic field (**H**_dc_ | |*x*), the ME optical response rotates the eigen polarization. The rotation angle of in-plane eigen polarization can be estimated from ${\varepsilon }_{xy}^{{{{{{\rm{S}}}}}}}$ in Fig. 2b. If we omit NOA and take into account Re[${\varepsilon }_{xy}^{{{{{{\rm{S}}}}}}}$], the rotation of eigen polarization from x and y axes is 8.8° at around 1 meV. In early works, the GB is discussed on the basis of the bare spectra of the optical rotation^11,15–17^. However, the rotation spectra are always modified by crystallographic linear birefringence, because the ME media with GB tend to be anisotropic as exemplified in this work; the in-plane polarization **P** and magnetization **M** necessarily cause the anisotropy.

**Fig. 3:**
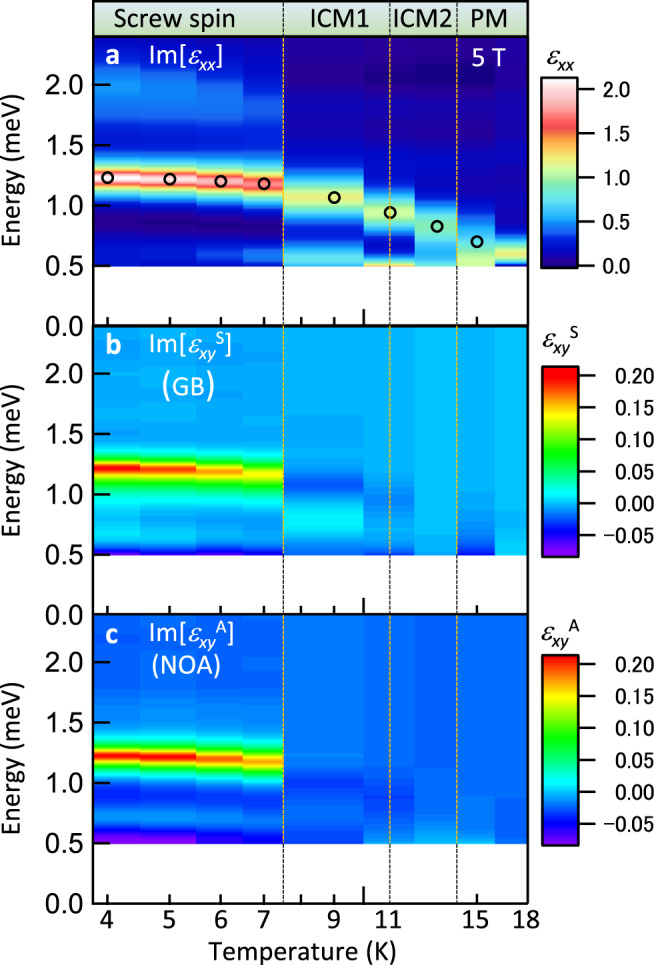
Correlation of optical activities with spin orders. Color-coded spectra of **a** electromagnon (Im[${\varepsilon }_{xx}$]), **b** GB (Im[${\varepsilon }_{xy}^{{{{{{\rm{S}}}}}}}$]), and **c** NOA (Im[${\varepsilon }_{xy}^{{{{{{\rm{A}}}}}}}$]) in the magnetic field (5 T, **H**_dc_||[110]). The open circles in **a** indicate the data acquisition temperatures. The GB and NOA emerge in the screw spin phase, manifesting that the ferroelectric and chiral screw spin order gives rise to the GB and NOA. (See Supplementary Note 3 for details of spectra).

To examine the correlation of these nonreciprocal gyrotropic effects with the screw spin order, the color-coded dielectric spectra of electromagnon (Im[${\varepsilon }_{xx}$], Fig. 3a), GB (Im[${\varepsilon }_{xy}^{{{{{{\rm{S}}}}}}}$], Fig. 3b), and NOA (Im[${\varepsilon }_{xy}^{{{{{{\rm{A}}}}}}}$], Fig. 3c) versus temperature are displayed. In the screw spin phase, both GB (Fig. 3b) and NOA (Fig. 3c) have peak structures on the electromagnon at 1.2 meV. The NOA and GB disappears upon the phase transition to ICM1 phases (∼8 K). This result is consistent with the fact that the spin order in the ICM1 phase loses the spin-driven **P** and chirality. In contrast, the resonance peak in Im[${\varepsilon }_{xx}$] (Fig. 3a) remains in higher temperature phases including PM phase. Thus, it is clearly demonstrated that the symmetry breaking via the screw spin order plays an essential role for GB as well as for NOA.

**Fig. 4:**
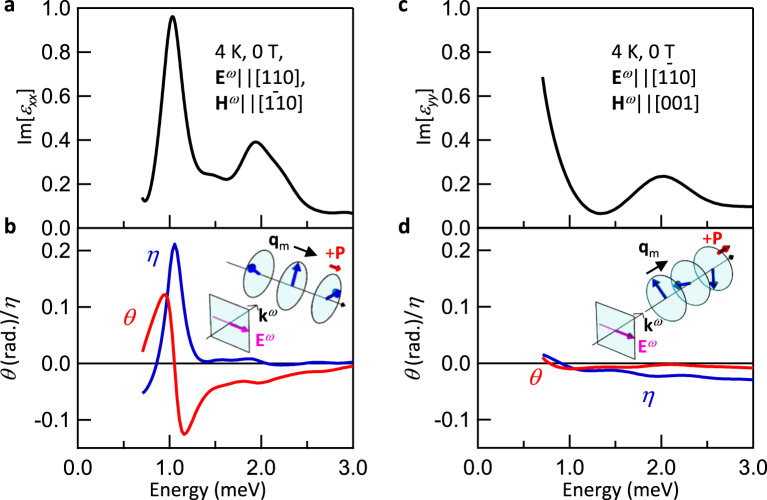
Polarization selection rule for NOA. **a** The spectrum of electromagnon (${\varepsilon }_{xx}$) obtained for (001) surface at 0 T in the screw spin phase. Experimental configuration (inset to (**b**)) is the same as that in Figs. 1, 2, and 3. **b** The rotation angle *θ* and ellipticity *η* at 0 T. The GB (${\varepsilon }_{xy}^{{{{{{\rm{S}}}}}}}$) is forbidden (see Fig. 2f) and the NOA (${\varepsilon }_{xy}^{{{{{{\rm{A}}}}}}}$) solely induces the optical rotation. **c** The spectrum for ${\varepsilon }_{yy}$ (*y*||[$1\bar{1}0$]) was obtained by using different samples with (110) surface at 0 T. The light polarization plane is perpendicular to **P**||[110] (inset to (**d**)). The observed spectrum is similar to ${\varepsilon }_{yy}$ obtained by using (001) surface (Fig. 2a). **d** The rotation angle *θ* and ellipticity *η* for (110) surface.

In the formalism in Eq. (1), the dynamical ME coupling ${\alpha }_{ii}$ is implicitly included in ${\varepsilon }_{xy}^{{{{{{\rm{S}}}}}}}$. The ${\varepsilon }_{xy}^{{{{{{\rm{S}}}}}}}$ representing GB is approximately expressed by ${\alpha }_{ii}$ as ${\varepsilon }_{xy}^{{{{{{\rm{S}}}}}}}$ ~ $\frac{\sqrt{{\varepsilon }_{xx}}+\sqrt{{\varepsilon }_{yy}}}{2}({\alpha }_{xx}-{\alpha }_{yy})$ under the assumption that the magnetic permeabilities are unity^10,11,31^. The interference between the electric and magnetic transition dipoles gives rise to the dynamical ME coupling, so that ${\alpha }_{ii}$ is proportional to <*g* | ${\mu }_{i}|e$><*e*|${m}_{i}$|*g* > ; ${\mu }_{i}$ and ${m}_{i}$ are the operators for electric and magnetic dipoles along *i* = *x*, *y*, *z*, and |*g* > and |*e* > denote the ground state and excited state, respectively^18^. On the other hand, the magnitude of NOA (${\varepsilon }_{xy}^{{{{{{\rm{A}}}}}}}$), which is time-reversal even, is proportional to −*i* < *g*|*μ*_i_|*e*><*e*|*m*_*i*_|*g* > ^31^. Accordingly, similar diagonal ME couplings are responsible for both GB and NOA. The electric transition dipole of electromagnon is polarized along *x* axis (${{{{{{\bf{E}}}}}}}^{\omega }||x$, Fig. 1e), suggesting that ${\alpha }_{xx}$ gives rise to the GB. In fact, for the (110) plane being perpendicular to the **P** (inset to Fig. 4d), the electromagnon resonance at 1.2 meV and optical rotation are both absent (Fig. 4c, d). In contrast, the optical rotation stemming only from NOA is observed at zero field in the original geometry (Fig. 1d), in which the (001) plane includes the **P**||*x* (Fig. 4a, b). Therefore, the coupled electric and magnetic transitions parallel to *x*, i.e., <*e*|*μ*_*x*_|*g* > and <*e*|*m*_*x*_|*g* > produces the NOA as well as the GB in this material.

In summary, we have demonstrated the nonreciprocal optical responses of GB and NOA by using the screw spin order in multiferroic CuFe_1-x_Ga_x_O_2_ (x = 0.035). The strong dynamical ME coupling of electromagnon exhibits the resonantly enhanced optical rotation arising from both GB and NOA. The spectra in the form of effective dielectric tensor obtained by using the terahertz time-domain polarimetry were exploited to distinguish GB and NOA. Accordingly, it has been concluded by symmetry arguments that the optical activity of GB can be viewed as the rotation of eigen linear polarization, which is contrasted with the conventional gyrotropic phenomena such as NOA having the circular birefringence/dichroism. The nonreciprocal response of GB revealed here expands the potential of optical functionality of condensed matter, enabling the control of the polarization state of light. The resonantly enhanced GB and NOA on the electromagnon applies to many multiferroic helimagnets in general. As demonstrated in Fig. 2f, the NOA and GB are independently controlled by the magnetic and electric fields, resulting in the full control of nonreciprocal optical rotation. Since the polarization rotation phenomena are associated with the phase change of light in matter, these optical activities are useful for tailoring the phase of light (Supplementary Note 4). Thus, the spin-driven GB and NOA can be exploited for the optically active devices based on multiferroics. Furthermore, the presently reported GB is not limited to the multiferroics, but also anticipated to show up in the axion electrodynamics in topological quantum materials^13,21^.

## Methods

Single crystal of CuFe_1-x_Ga_x_O_2_ (x = 0.035) was grown by the floating-zone method. For the optical measurement, the (001) and (110) surfaces were used. The typical sample dimension is 3 mm × 3 mm × 1 mm. To manipulate the ferroelectric (chiral) domain for (001) plane sample, the external magnetic field (5 T) and electric field (180 kV/m) along [110] were applied while cooling the sample. Once the single ferroelectric domain is formed, the P remains intact even without an electric field and magnetic field. The external electric field is absent during the optical measurements. To obtain the single ferroelectric domain for (110) surface (Fig. 4c, d), we use the 120° flop of **q**_m_ by the rotation of magnetic field in this material^9,29^.

Terahertz time-domain polarimetry was carried out in the magnetic field applied parallel to [110], which enables us to determine the effective dielectric tensor in photon energy from 0.7 to 3 meV. The laser pulses with a photon energy of 1.55 eV, pulse duration of 100 fs, and repetition rate of 80 MHz were used. The laser pulses were split into two paths to generate and detect the terahertz pulses. The terahertz pulses were emitted and detected by the photoconductive antennas. The polarization of incident light is set to [110] (*x* axis) or [$1\bar{1}0$] (*y* axis) by using the wire grid polarizer (Fig. 1d). The detailed specifications of the terahertz polarimeter is described in Supplementary Note 5. The optical rotation of the transmitted light was measured by using the wire grid polarizer set to ±45 º with respect to the *x* axis (Fig. 1d). To analyze the terahertz waveform, the Fourier transformation of the transmitted terahertz pulses, *E*_+45°_(*ω*) and *E*_-45°_(*ω*), were obtained. The rotation angle *θ* and ellipticity *η* in Fig. 1f are defined as

$$\frac{\sin \,\theta +i\,\eta \cos \,\theta }{\cos \,\theta -i\,\eta \sin \,\theta }=\frac{{E}_{+45^\circ }(\omega )-{E}_{+45^\circ }(\omega )}{{E}_{+45^\circ }(\omega )+{E}_{+45^\circ }(\omega )}.$$

We measured four transmittance waveforms; *E*_+45°_, *E*_−45°_ for each incident light polarized along *x* and *y* axes (Fig. 1d). These in-plane dielectric spectra composed of ${\varepsilon }_{xx}(\omega )$, ${\varepsilon }_{yy}(\omega ),\,{\varepsilon }_{xy}(\omega )$, and ${\varepsilon }_{yx}(\omega )\,$were calculated from these four waveforms and references by a direct procedure. To eliminate the leakage signal of wire grid polarizer, we antisymmetrized the raw waveform of rotational signal for +**P** and −**P**, because the optical rotations of both GB and NOA show the sign change under the reversal of **P**. To discuss the GB and NOA independently, the off-diagonal spectra, ${\varepsilon }_{yx}(\omega )$ and ${\varepsilon }_{xy}(\omega )$, were symmetrized or antisymmetrized, resulting in ${\varepsilon }_{xy}^{{{{{{\rm{S}}}}}}}(\omega )$ and ${\varepsilon }_{xy}^{{{{{{\rm{A}}}}}}}(\omega )$ respectively. The example of (anti)symmetrization of off-diagonal spectra is described in detail in Supplementary Note 6.

## Supplementary information

Supplementary Information

## Data Availability

Source data are provided with this paper. All other data that support the plots within this paper are available from the corresponding authors upon reasonable request. The data that support the plots of this study are available from the corresponding author upon reasonable request. Source data are provided with this paper.

## Source data

Source Data
