# Supplementary material for: Enhanced gyrotropic birefringence and natural optical activity on electromagnon resonance in a helimagnet
Source: Nat Commun. 2021 Nov 18;12:6674. doi: 10.1038/s41467-021-26953-x (PMC8602373; doi:10.1038/s41467-021-26953-x)
Supplement: Supplementary file 1 — Supplementary Information [file 41467_2021_26953_MOESM1_ESM.pdf]

Supplementary information:
Enhanced gyrotropic birefringence and natural optical activity on electromagnon resonance in a helimagnet

S. Iguchi[1]*, R. Masuda[1], S. Seki[1,2], Y. Tokura[1,3,4], and Y. Takahashi[1,3]*

[1]Department of Applied Physics and Quantum Phase Electronics Center (QPEC), The University of Tokyo, Tokyo 113-8656, Japan.

[2]Institute of Engineering Innovation, The University of Tokyo, Tokyo 113-0032, Japan

[3]RIKEN Center for Emergent Matter Science (CEMS), Wako, Saitama 351-0198, Japan.

[4]Tokyo College, University of Tokyo, Tokyo 113-8656, Japan.

*E-mail: wigtywigty@yahoo.co.jp; youtarou-takahashi@ap.t.u-tokyo.ac.jp

Supplementary Note 1: Off-diagonal spectra of effective dielectric spectra

The off-diagonal spectra ($\varepsilon_{xy}(\omega)$ and $\varepsilon_{yx}(\omega)$) of effective dielectric tensor for respective ME geometries including (**a**) +**M**, +**P**, (**b**) +**M**, −**P**, (**c**) −**M**, +**P**, (**d**) −**M**, −**P** are displayed in Fig. S1. The spectra are reversed in sign with the sign reversal of **P** (Fig. S1a-b), because both GB and NOA have odd parity for the reversal of **P**. On the other hand, the different-shape spectra were observed for the sign reversal of **M** (Fig. S1a and S1c), because the GB and NOA have odd and even parity for **M**, respectively. The (anti-)symmetric part of dielectric spectra are derived as $\varepsilon_{xy}^{\mathrm{S}} = \frac{\varepsilon_{xy}+\varepsilon_{yx}}{2}$ ($i\varepsilon_{xy}^{\mathrm{A}} = \frac{\varepsilon_{xy}-\varepsilon_{yx}}{2}$) for respective ME geometries, as shown in Fig. 2b-e of the main text.

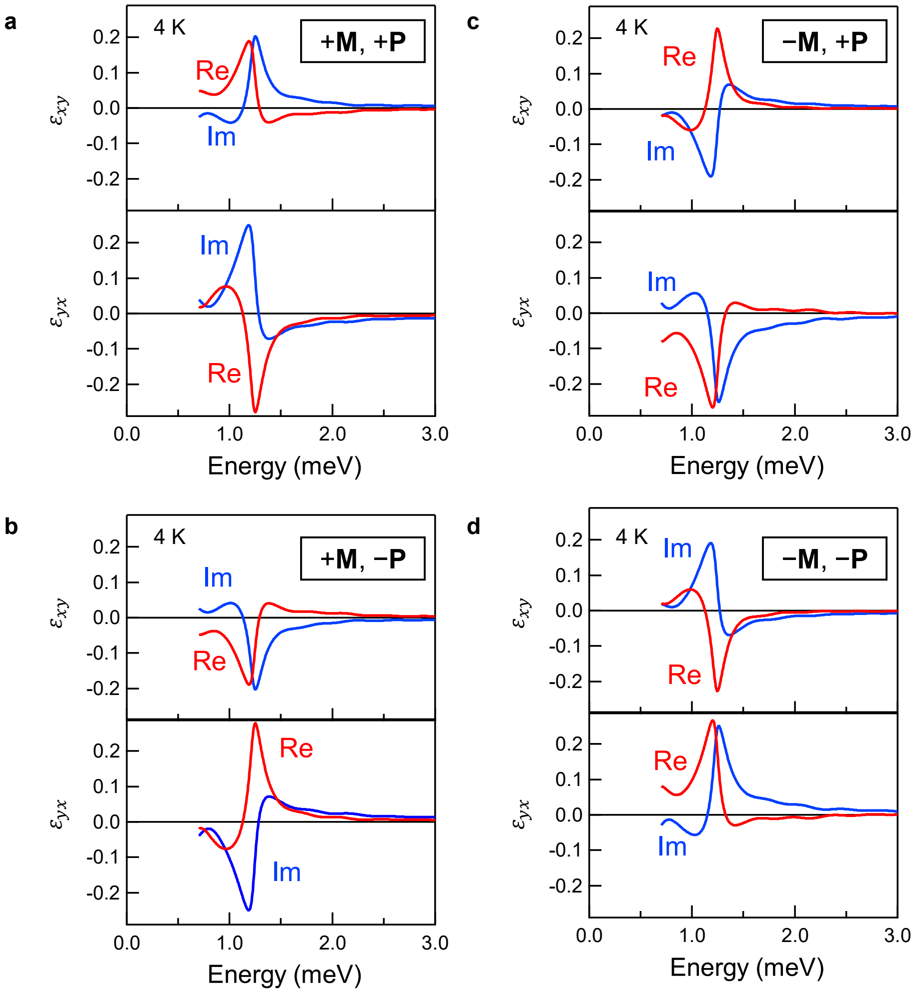

Fig. S1| The off-diagonal spectra of effective dielectric tenosr, $\varepsilon_{xy}(\omega)$ and $\varepsilon_{yx}(\omega)$, at 4 K, 5 T for (**a**) +**M**, +**P**, (**b**) +**M**, −**P**, (**c**) −**M**, +**P**, (**d**) −**M**, −**P**. Red and blue lines indicate the real (Re) and imaginary (Im) parts, respectively.

Supplementary Note 2: Magnetic field dependence of GB and NOA

The magnetic field dependence of GB ($\varepsilon_{xy}^{\mathrm{S}}$) and NOA ($\varepsilon_{xy}^{\mathrm{A}}$) for +**P**, or equivalently γ_{m}=+, are displayed in Fig. S2. The GB ($\varepsilon_{xy}^{\mathrm{S}}$) changes the sign with the reversal of magnetic field (Fig. S1a and b). In contrast, the spectra for NOA ($\varepsilon_{xy}^{\mathrm{A}}$) is preserved for the reversal of the magnetic field (Fig. S1c and d). These results are summarized in Fig. 2f of the main text. As shown in Fig. 2a, the resonance energy of electromagnon (ε_{xx}) increases as the magnetic field is increased. In accord with the hardening of electromagnon, the energy shift of GB ($\varepsilon_{xy}^{\mathrm{S}}$, Fig. S2b) and NOA ($\varepsilon_{xy}^{\mathrm{A}}$, Fig. S2d) is observed. The peak magnitude of GB (Im[$\varepsilon_{xy}^{\mathrm{S}}$], Fig. S2b) increases with the increase of magnetic field, whereas the magnitude of NOA shows little magnetic field dependence.

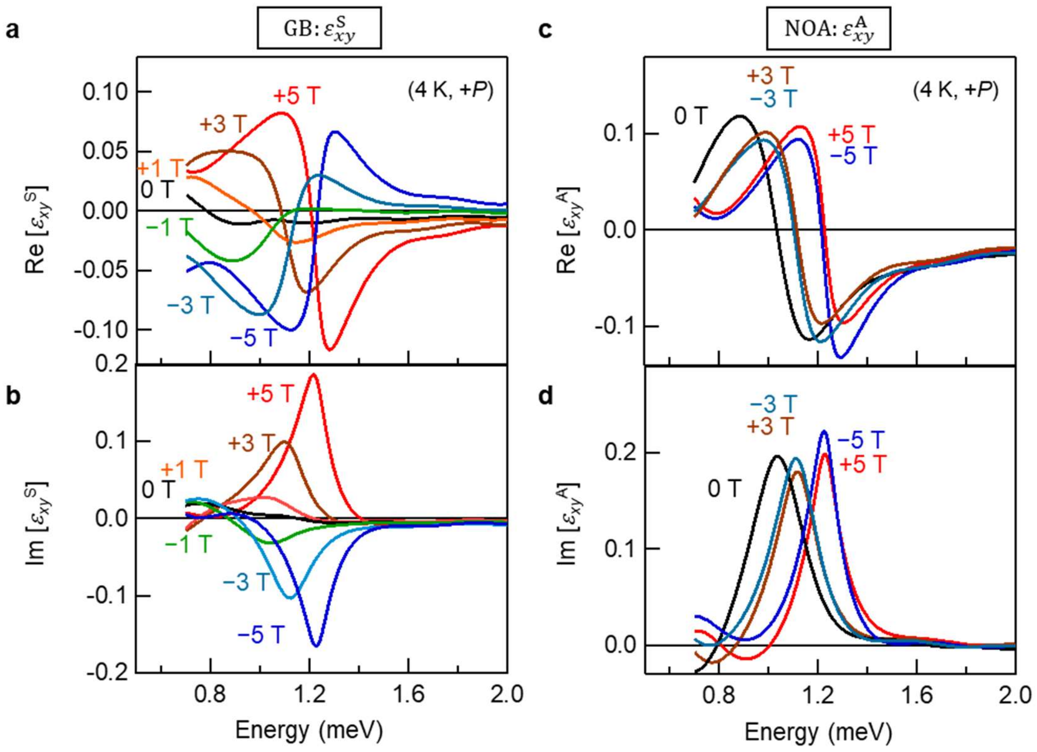

Fig. S2| Magnetic field dependence of complex spectra for GB ($\varepsilon_{xy}^{\mathrm{S}}$); (**a**) real part and (**b**) imaginary part. Magnetic field dependence of complex spectra for NOA ($\varepsilon_{xy}^{\mathrm{A}}$); (**c**) real part and (**d**) imaginary part. All the measurements were done at 4 K while keeping the sign of **P** and equivalently the sign of γ_{m}.

Supplementary Note 3: Temperature dependence of electromagnon, GB and NOA

The temperature dependence of the electromagnon (ε_{xx}), GB ($\varepsilon_{xy}^{\mathrm{S}}$) and NOA ($\varepsilon_{xy}^{\mathrm{A}}$) is summarized in the color-coded spectra in Fig. 3, whose spectra are displayed in Fig. S3 in addition to the spectra of ε_{yy}. With increasing temperature, the electromagnon resonance at 1.2 meV shifts toward the lower energy and their magnitude decreases in the screw spin phase (Fig. S3a, 4 K, 6 K, 7 K). The peak structure is observed even in the higher-temperature phases (9 K, 13 K). On the other hand, the resonance peak is not discerned for the ε_{yy} (Fig. S3b). The GB ($\varepsilon_{xy}^{\mathrm{S}}$, Fig. S3c) and NOA ($\varepsilon_{xy}^{\mathrm{A}}$, Fig. S3d) show the clear resonance in the low-temperature screw spin phase (4 K, 6 K, 7 K), while these resonance peaks disappear in the ICM1 (9 K) and ICM2 (13 K) phases.

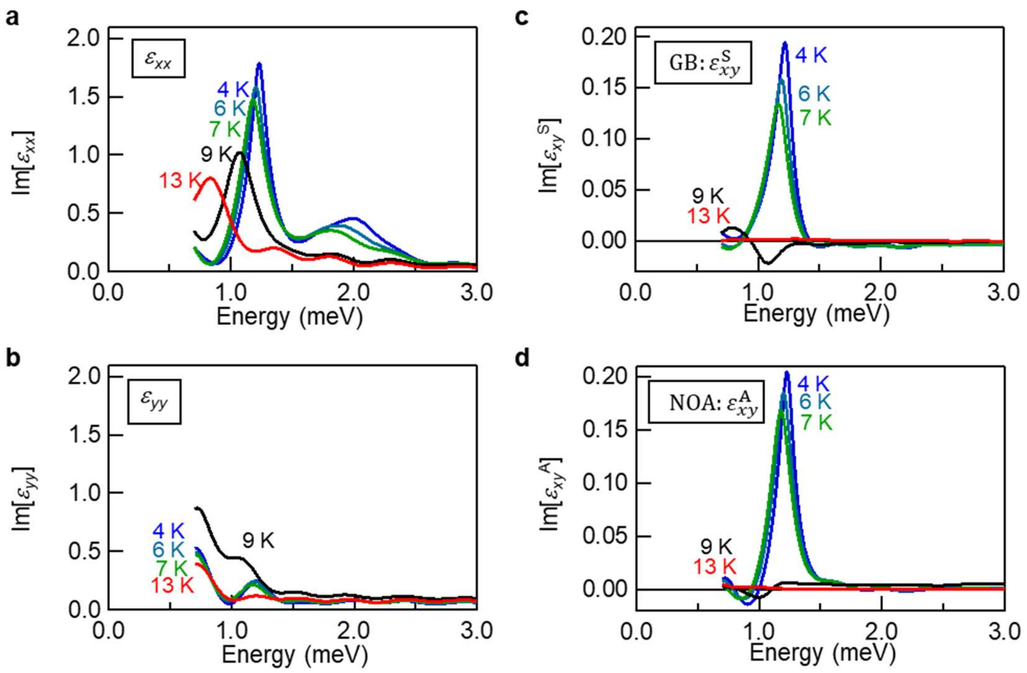

Fig. S3| Temperature dependence of (**a**) electromagnon (ε_{xx}), (**b**) ε_{yy}, (**c**) GB ($\varepsilon_{xy}^{\mathrm{S}}$) and (**d)** NOA ($\varepsilon_{xy}^{\mathrm{A}}$). These spectra are summarized in the color-coded spectra in Fig. 3 in the main text.

Supplementary Note 4: Raw terahertz waveforms

The example of raw terahertz waveforms is displayed in Fig. S4. In addition to the parallel components in Fig. S4(b), the rotatory components were observed owing to the optical rotation of GB and NOA (Fig. S4(c-e)). Only the NOA rotates the light polarization at 0 T (black line in Fig, S4(c)). Both NOA and GB cause the optical rotation at 5 T, and the mixing of two different gyrotropy results in the change of phase and amplitude of terahertz field (red line in Fig. S4(c)). The reversal of P changes the sign of both NOA and GB, so that the oscillation phase is reversed (Fig. S4(d)). The reversal of magnetic field causes the sign change of GB, while the sign of NOA is preserved. Thus, the rotatory terahertz field shows the phase shift (Fig. S4(e)).

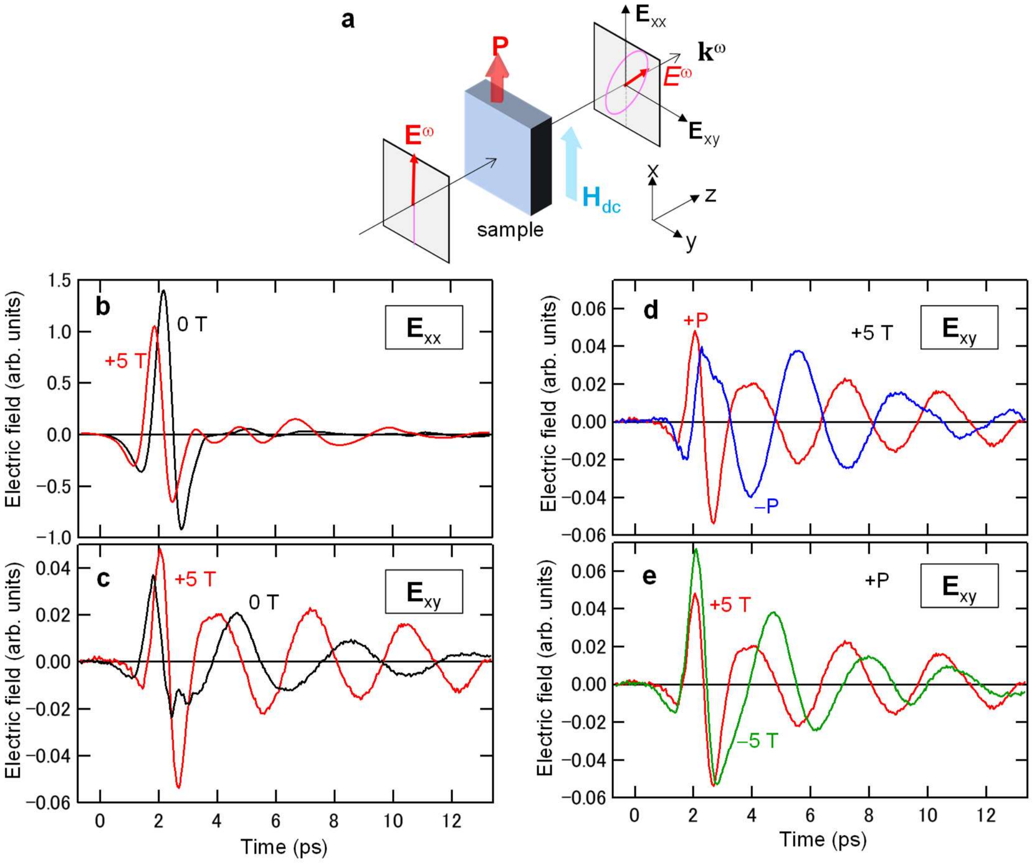

Fig. S4| a. Experimental configuration of terahertz polarimetry. **b.** Terahertz field after the sample with the light polarization parallel to the incident terahertz wave at 4 K. **c**. Rotatory components of terahertz field after the sample at zero magnetic field and at 5 T. **d**. Rotatory components of terahertz field at +5 T under the reversal of **P**. **e**. Rotatory components of terahertz field for +**P** under the reversal of magnetic field (**H**$_{dc}$).

Supplementary Note 5: Specification of terahertz time-domain polarimeter

We measured the optical rotation by using the terahertz time-domain polarimetry. The terahertz fields for parallel polarizers and crossed Nicols (Fig. S5(b)) without sample are displayed in Fig. S5(a). The finite signal observed under crossed Nicols indicates the leakage of wire grid polarizers. The power spectrum of terahertz field for parallel polarizers shows that the signal noise ratio of our equipment is about 10^{-6} (Fig. S5(c)). The power transmittance in Fig. S5(d), which is calculated by the power spectrum under crossed Nicols divided by that for parallel polarizers, shows the extinction ratio of wire grid polarizer used in our measurements. At around 2 meV, the extinction ration is as large as 10^{-4}. The constant leakage signal can be eliminated by antisymmetrizing the rotatory components of terahertz field (see method).

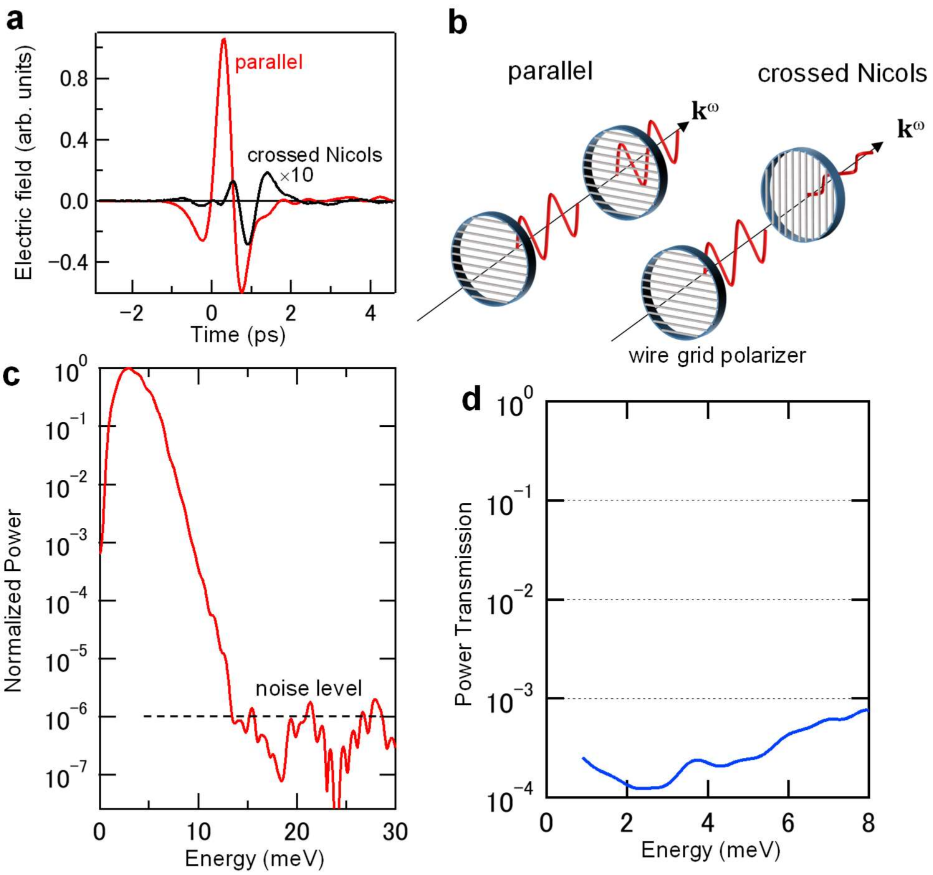

Fig. S5| a. Terahertz fields obtained for parallel polarizers (left in (b)) and crossed Nicols (right in (b)). **c**. Power spectrum of terahertz field for the parallel polarizers. **d**. Power transmission under crossed Nicols.

Supplementary Note 6: Example of data analysis

We calculated the spectra representing the GB ($\varepsilon_{xy}^{\mathrm{S}}$) and NOA ($\varepsilon_{xy}^{\mathrm{A}}$) shown in Figs. 2-4 in the main text as follows. At each measurement condition distinguished by temperature, magnetic field and sign of **P**, we obtained effective dielectric spectra in the form of 2×2 matrix. For example, the effective dielectric tensor at 4 K, +5 T with +P is displayed in Fig. S6(a). In addition to the diagonal components (ε_{xx} and ε_{yy}), the off-diagonal components (ε_{xy} and ε_{yx}) have the resonance structure owing to the GB and NOA. The symmetric part of off-diagonal component ($\varepsilon_{xy}^{\mathrm{S}}$) representing GB is calculated from the spectra of ε_{xy} and ε_{yx} as shown in Fig. S6(b). Similarly, the antisymmetric part ($\varepsilon_{xy}^{\mathrm{A}}$) representing NOA is calculated from ε_{xy} and ε_{yx} as shown in Fig. S6(c). The spectra of $\varepsilon_{xy}^{\mathrm{S}}$ and $\varepsilon_{xy}^{\mathrm{A}}$ summarized in main manuscript text (Fig. 2-4) were calculated in the same way at each experimental condition.

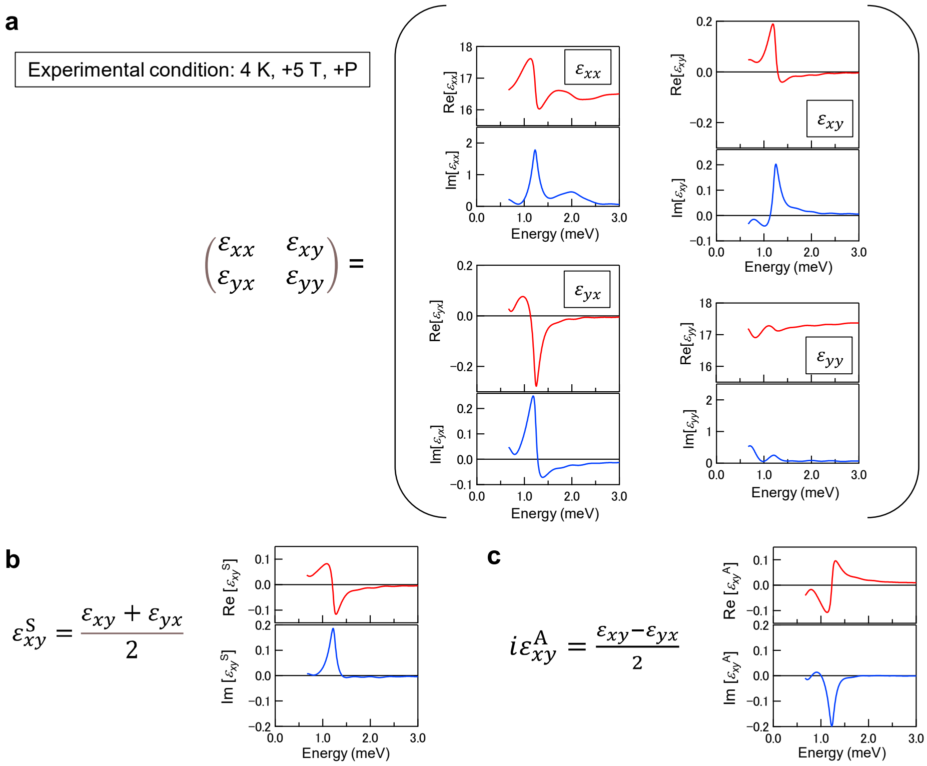

Fig. S6| a. Effective dielectric spectra in the form of 2×2 matrix at 4 K, +5 T and for +P. **b**. Derivation of symmetric term ($\varepsilon_{xy}^{\mathrm{S}}$) from off-diagonal spectra in (a). **c**. Derivation of antisymmetric term ($\varepsilon_{xy}^{\mathrm{A}}$) from off-diagonal spectra in (a).
